# Reduced Lipid Peroxidation Predicts Unfavorable Prognosis in Hepatocellular Carcinoma, but Not Intrahepatic Cholangiocarcinoma

**DOI:** 10.3390/biomedicines11092471

**Published:** 2023-09-06

**Authors:** Tiemo Sven Gerber, Hagen Roland Witzel, Arndt Weinmann, Fabian Bartsch, Mario Schindeldecker, Peter R. Galle, Hauke Lang, Wilfried Roth, Dirk Andreas Ridder, Beate Katharina Straub

**Affiliations:** 1 Institute of Pathology, University Medical Center of the Johannes Gutenberg University, 55131 Mainz, Germany; tiemo.gerber@unimedizin-mainz.de (T.S.G.); hagen.witzel@unimedizin-mainz.de (H.R.W.); mario.schindeldecker@unimedizin-unimedizin.de (M.S.); wilfried.roth@unimedizin-mainz.de (W.R.); d.ridder@pathologie-ludwigshafen.de (D.A.R.); 2 Department of Internal Medicine, University Medical Center of the Johannes Gutenberg University, 55131 Mainz, Germany; arndt.weinmann@unimedizin-mainz.de (A.W.); peter.galle@unimedizin-mainz.de (P.R.G.); 3 Department of General, Visceral and Transplant Surgery, University Medical Center of the Johannes Gutenberg University, 55131 Mainz, Germany; fabian.bartsch@unimedizin-mainz.de (F.B.); hauke.lang@unimedizin-mainz.de (H.L.); 4 Tissue Biobank, University Medical Center of the Johannes Gutenberg University, 55131 Mainz, Germany

**Keywords:** 4-HNE, reactive oxygen species, hepatocellular carcinoma, intrahepatic cholangiocarcinoma, biomarker, liver cirrhosis

## Abstract

Primary liver cancer, including hepatocellular carcinoma (HCC) and intrahepatic cholangiocarcinoma (iCCA), remains a significant contributor to cancer-related mortality worldwide. Oxidative stress and lipid peroxidation play a key role in chronic liver diseases and have been shown to be pivotal for tumor initiation and progression. 4-hydroxy-nonenal (4-HNE), one of the major mediators of oxidative stress and a well-established biomarker for lipid peroxidation, can act as a signal transducer, inducing inflammation and exerting carcinogenic effects. However, the role of 4-HNE in primary liver cancer remains poorly explored. In this study, we investigated 4-HNE levels in 797 liver carcinomas, including 561 HCC and 236 iCCA, by immunohistochemistry. We then correlated 4-HNE levels with comprehensive clinical data and survival outcomes. In HCC, lower expression levels of 4-HNE were associated with vascular invasion, a high tumor grade, a macrotrabecular-massive HCC subtype, and poor overall survival. Concerning iCCA, large duct iCCA showed significantly higher 4-HNE levels when compared to small duct iCCA. Yet, in iCCA, 4-HNE levels did not correlate with known prognostic parameters or survival outcomes. To conclude, in HCC but not in iCCA, low amounts of 4-HNE predict unfavorable survival outcomes and are associated with aggressive tumor behavior. These findings provide insights into the role of 4-HNE in liver cancer progression and may enable novel therapeutic strategies.

## 1. Introduction

Liver cancer is the fourth most common cause of cancer-related death worldwide, with hepatocellular carcinoma (HCC) being the most frequent tumor type, followed by intrahepatic cholangiocarcinoma (iCCA) [1]. Both HCC and iCCA are evaluated using the Union for International Cancer Control (UICC) staging system. For HCC, clinical decision-making and prognosis assessment are performed using supplementary tumor staging systems. such as the Barcelona Clinic Liver Cancer (BCLC), Hong Kong Liver Cancer (HKLC), or Japan Integrated Staging (JIS) staging system. These staging systems allow robust stratification into distinct prognostic groups. Nonetheless, the inherent variability observed in the clinical course of individual HCC patients underscores the need for further improvement in the evaluation of prognosis [2]. Unlike for HCC, no generally accepted staging systems have been tailored for iCCA.

Oxidative stress, characterized by an imbalance between the production of reactive oxygen species (ROS) and antioxidant systems, can lead to ROS interaction with cellular macromolecules, particularly DNA, membrane phospholipids, and proteins, thereby causing cellular damage and dysfunction [3]. ROS, generated during normal cellular processes like mitochondrial metabolism, also serves as a signaling molecule, promoting cell proliferation and activating survival pathways at low levels. Furthermore, high ROS levels can cause DNA damage, promoting mutagenesis, and have therefore been implicated in tumor initiation and progression [4]. On the other hand, high levels of ROS may also result in cell death and senescence [5]. Tumor cells are therefore thought to equilibrate ROS levels to avoid the detrimental effects of oxidative stress. In this line, it has recently been demonstrated that not only ROS but also antioxidant pathways may be involved in cancer initiation and progression [6].

Lipid peroxidation is one of the cellular consequences of uncontrolled ROS elevation. Among the aldehydes produced during lipid peroxidation, 4-hydroxy-nonenal (4-HNE) is one of the best-studied molecules [7,8]. It is considered the major biomarker of lipid peroxidation and is a signaling molecule involved in the regulation of transcription factors associated with oxidative stress [9]. 4-HNE becomes apparent as a pivotal pathological target in various diseases, capable of inducing both inflammation and apoptosis while also deactivating key proteins. Consequently, 4-HNE is regarded as a molecule of substantial concern. The authors of [10] report that 4-HNE not only mediates oxidative stress but has also been implied as a signaling molecule exerting several biological effects upon high ROS levels. The study conducted by Podszun et al. exemplifies the reliability and robustness of immunohistochemical evaluation of 4-HNE protein adducts as an approach for visualizing and assessing lipid peroxidation in liver tissue [11]. Recently, it has been demonstrated that there are characteristic changes in the lipid profile of HCC cells that are paralleled by decreases in 4-HNE levels, which may be beneficial for tumor growth and progression [12]. Additionally, 4-HNE has been implicated in tumor initiation in HCC, both in animal models and in humans [13].

ROS generated under inflammatory conditions play a crucial role in iCCA as well, contributing to the formation of oxidative DNA adducts and promoting inflammation-mediated carcinogenesis [14]. While the effects of ROS on iCCA have been extensively investigated, there is currently no available data regarding the correlation between 4-HNE and iCCA.

The present immunohistochemical study investigates 4-HNE levels in a large cohort of patients with intrahepatic cholangiocarcinoma and hepatocellular carcinoma and correlates 4-HNE expression with clinical data and survival outcomes.

## 2. Materials and Methods

### 2.1. Patient Cohort

A total of 294 paraffin-embedded formalin-fixed (FFPE) samples from 236 iCCA patients and 871 FFPE samples from 561 HCC patients were collected from routine histopathology cases seen at the Institutes of Pathology, University Medical Center Mainz, between 2006 and 2020. Tissue microarrays (TMAs) with cores of 1–2 mm were constructed. Each patients‘ set of samples encompassed the primary tumor, additional tumor foci, corresponding normal tissue, precursor lesions, relapse tumors, and lymph node or distant metastases, if present.

### 2.2. Immunohistochemistry

Immunohistochemistry (IHC) was carried out on 4 µm-thick histological sections. Antigen retrieval was undertaken with Tris/EDTA buffer, pH 9 (Dako, Santa Clara, California, #8024), and cell conditioning solution 1 (Roche, Mannheim, Germany, #950-124). TMA slides were incubated with a mouse monoclonal anti-4-hydroxynonenal antibody (Abcam, Berlin, Germany; ab48506) and a monoclonal anti-glutamine synthetase antibody (Roche, Mannheim, Germany; 760–4898). Immunohistochemistry was undertaken with an automated staining system (DAKO Autostainer Plus, Agilent Technologies, Santa Clara, CA, USA) with the Dako EnVision FLEX staining method (Agilent Technologies). Staining procedures were performed as indicated by the manufacturers’ guidelines. Stained slides were digitized using a whole slide scanner set at 40× magnification (Nanozoomer, Hamamatsu Photonics, Hamamatsu, Japan). Evaluation of staining intensity involved the application of the semiquantitative immunoreactivity score (IRS), calculated by multiplying the staining intensity (ranging from 0 to 3) by the extent of tumor involvement (ranging from 0 to 4), resulting in score values spanning from 0 to 12 [15]. Glutamine synthetase (GS) overexpression has been defined as a uniform staining pattern of similar intensity as observed in zone 3 hepatocytes of non-neoplastic liver tissue. The immunohistochemical evaluation was executed by experienced pathologists (T.S.G. and D.A.R.) and supervised by an expert hepatopathologist (B.K.S.).

### 2.3. Statistical Analysis

All statistical analyses were carried out using the R environment for statistical computing (version 4.3.0) [16]. The non-parametric Mann–Whitney U test was employed to compare two independent groups characterized by non-normally distributed continuous variables or ordinal data. When examining the concordance of distributions within two dependent samples, the Wilcoxon signed-rank test was employed to assess whether they were drawn from populations exhibiting identical distributions. To mitigate the impact of multiple testing and curtail the risk of false positives or Type I errors, corrections according to the Benjamini–Hochberg method were implemented. Dichotomization of 4-HNE and AFP protein expression was conducted utilizing the Charité cut-off finder functions, which enabled the establishment of a significant demarcation between high and low protein expression levels predicated on survival outcome [17]. The assessment of overall survival entailed the calculation of the time span between initial diagnosis and death, irrespective of etiology or the last follow-up, analyzed through the Kaplan–Meier method, with disparities evaluated using the log-rank test. Uni- and multivariate Cox regression analyses were executed using the function coxph from the R package survival [18,19]. Adjusted *p* values were reported exclusively if divergent from the non-adjusted values to ensure clarity in the presentation. To examine associations between pairs of variables, the Spearman rank-order correlation coefficient was computed for non-metric and non-normally distributed data, while the Pearson correlation coefficient was employed for metric and normally distributed data.

## 3. Results

### 3.1. Association of Low Intratumoral Amounts of 4-HNE with Increased Vascular Invasion and Poor Tumor Differentiation in HCC Patients

**Figure 1:**
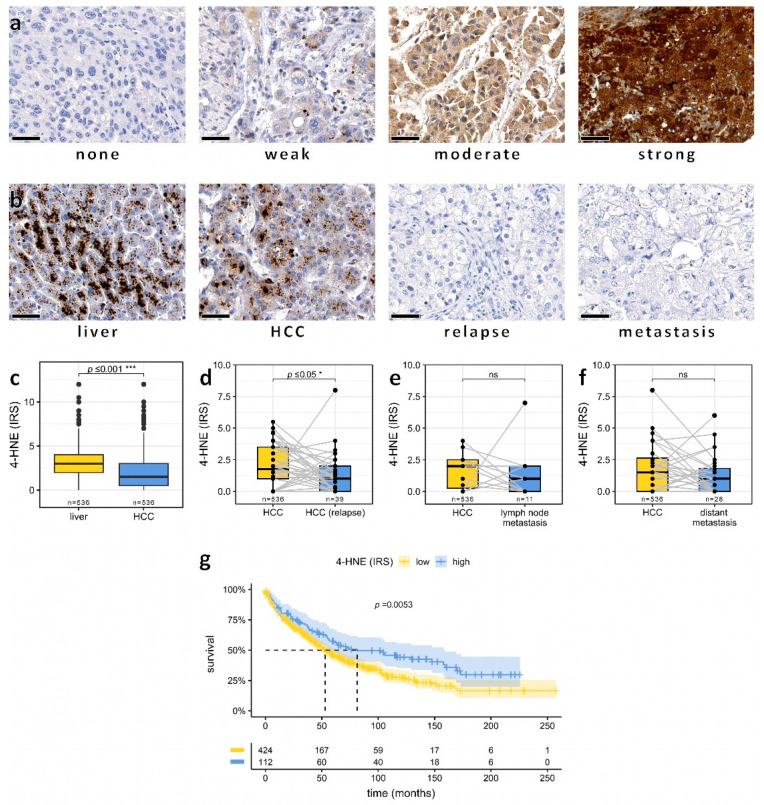
Low intratumoral amounts of 4-HNE predict unfavorable outcomes in HCC patients. (**a**) Representative immunohistochemistry images demonstrating 4-HNE staining levels categorized as none, weak, moderate, and strong. Bars: 50 µm. (**b**) Examples of representative 4-HNE staining patterns in normal hepatocytes, hepatocellular carcinoma (HCC), relapsed HCC, and HCC metastasis. Boxplot illustrating the reduction of the 4-HNE immunoreactive score (IRS) from (**c**) surrounding liver tissue to primary HCC, (**d**) primary HCC to HCC relapses, (**e**) HCC to lymph node metastasis, and (**f**) HCC to distant metastases. (**g**) Kaplan–Meier plot of overall survival in HCC patients stratified by 4-HNE IRS levels. If a *p* value is less than 0.05, it is flagged with one star (*). If a *p* value is less than 0.001, it is flagged with three stars (***). No significance is labeled as “ns”.

To analyze the effects of 4-HNE protein expression on HCC progression and outcome, we used a well-characterized tissue microarray with 871 HCC from 561 patients with detailed clinical data [20,21,22]. 4-HNE immunohistochemistry was performed to evaluate 4-HNE staining using the IRS score. In normal liver tissue (*n* = 536), 4-HNE was strongly detected in the cytoplasm of hepatocytes, displaying a granular staining pattern. However, in cells with primary HCC, 4-HNE levels were significantly lower (Figure 1a–c). Relapse tumors (*n* = 39) showed a further reduction of 4-HNE compared to the respective primary HCC (Figure 1d). Moreover, there was a trend towards lower 4-HNE levels in lymph nodes (*n* = 11) and distant metastases (*n* = 28) compared to primary HCC (*n* = 536), although this trend did not reach statistical significance (Figure 1e,f). Survival analyses revealed that the subgroup with low 4-HNE expression levels exhibited significantly worse survival outcomes than the group with high 4-HNE levels (Figure 1g).

### 3.2. Association of Low Intratumoral Amounts of 4-HNE with Increased Vascular Invasion and Poor Tumor Differentiation in HCC Patients

**Figure 2:**
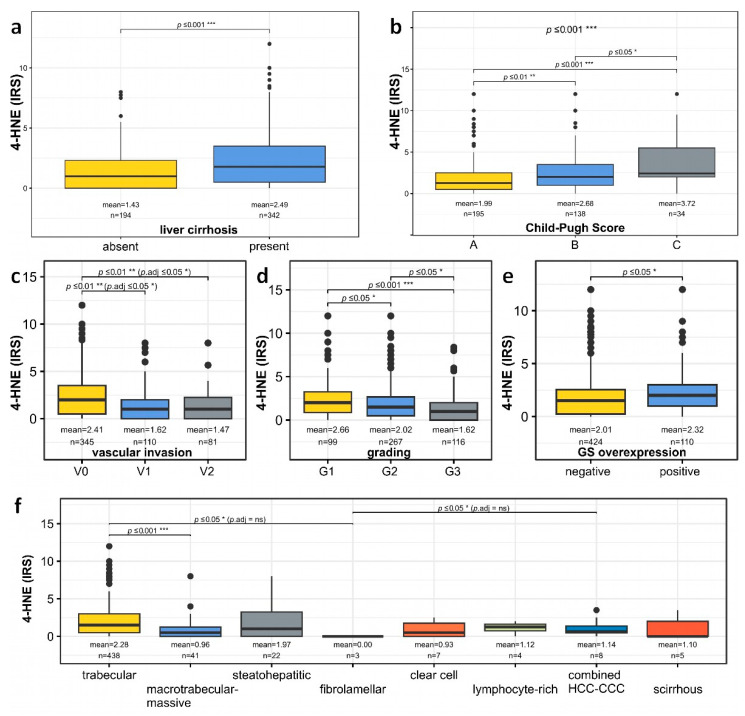
Boxplots comparing 4-HNE levels in different HCCs. 4-HNE levels in (**a**) patients with and without liver cirrhosis and, if present, in (**b**) patients with different Child–Pugh scores. (**c**) Distribution of 4-HNE levels in HCC patients with no (V0), microscopic (V1), or macroscopic (V2) vascular invasion. (**d**) HCC patients are categorized based on tumor differentiation (G1: well differentiated, G2: moderately differentiated, G3: poorly differentiated). (**e**) HCC patients with and without glutamine synthetase (GS) overexpression. (**f**) Illustration of 4-HNE levels among various HCC subtypes. If a *p* value is less than 0.05, it is flagged with one star (*). If a *p* value is less than 0.01, it is flagged with 2 stars (**). If a *p* value is less than 0.001, it is flagged with three stars (***). No significance is labeled as “ns”.

To evaluate the relationship of 4-HNE with different established prognostic factors, low and high 4-HNE levels were correlated with the BCLC staging system [23], vascular invasion [24,25], larger tumor diameter [26], Child–Pugh Score [27], and macrotrabecular-massive growth pattern [28]. The patient group with lower intratumoral amounts of 4-HNE showed significantly higher patient age and larger tumor diameter, more frequent vascular invasion, and a more frequent macrotrabecular-massive growth pattern, but less frequent liver cirrhosis and, if cirrhosis was present, a lower Child–Pugh score (Table 1, Figure 2a,b).

In line with these findings, further analysis also demonstrated a significant reduction of intratumoral 4-HNE levels in HCC with vascular invasion, a higher tumor grade, GS overexpression, and a macrotrabecular-massive subtype (Figure 2c–f). Age at diagnosis and 4-HNE IRS of non-neoplastic liver tissue were not significantly correlated, *r_s_* (534) = 0.118, *p* = 0.785.

### 3.3. Multivariate Analysis Confirms Lower 4-HNE Levels as an Independent Predictor of Adverse Outcomes in HCC

**Figure 3:**
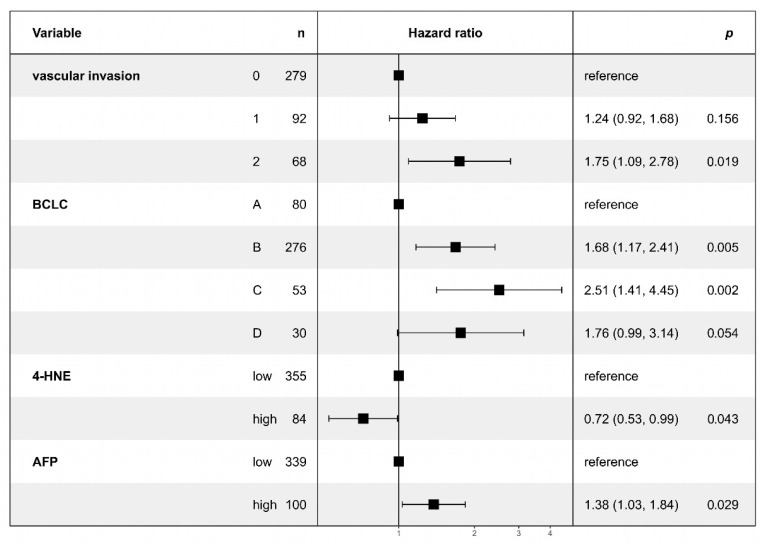
Multivariate Cox regression analysis of overall survival on prognostic factors in hepatocellular carcinoma (HCC) patients. The squares represent the hazard ratios, and the horizontal lines show the confidence intervals. The analysis includes vascular invasion (0: none, 1: microscopic, 2: macroscopic), Barcelona Clinic Liver Cancer (BCLC) stage (A, B, C, D), immunohistochemically detected 4-HNE levels (low, high), and serum alpha-fetoprotein (AFP) levels (low, high; cutoff: 240 ng/mL). Note the significantly decreased risk of adverse outcomes in patients with high 4-HNE levels.

To demonstrate the correlation between different prognostic factors, we performed a Cox regression analysis for overall survival (Figure 3). Multivariate analysis confirmed the prognostic significance of 4-HNE in HCC. Patients with low 4-HNE levels demonstrated poorer outcomes, in parallel with established factors such as the presence of vascular invasion, higher BCLC stage, and a higher serum AFP. No evidence of reliable collinearity between BCLC stage and vascular invasion was detected in our analysis, with the highest variance inflation factor observed being 3.18.

In conclusion, our findings indicate that 4-HNE immunohistochemistry may independently predict overall survival, with low 4-HNE expression being associated with poorer survival outcomes.

### 3.4. No Significant Association between 4-HNE Levels and Patient Survival in iCCA

**Figure 4:**
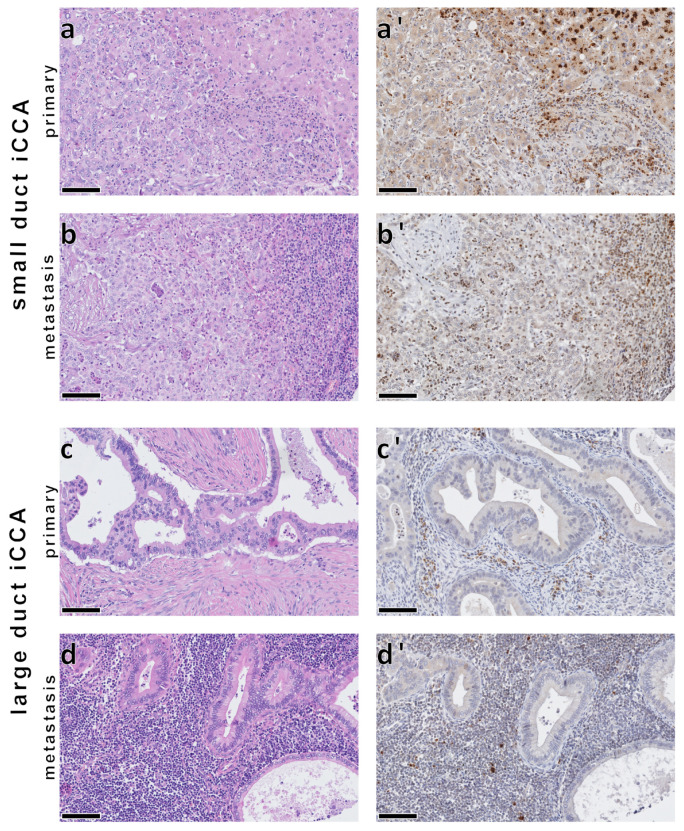
4-HNE immunohistochemistry in intrahepatic cholangiocarcinomas (iCCAs) of small (**a**,**a’**) and large (**c**,**c’**) duct types and their respective lymph node metastases (**b**,**b’**,**d**,**d’**). iCCAs demonstrate an overall weak staining reaction to 4-HNE, which is contrasted by strong staining intensity in the adjacent non-neoplastic hepatocytes ((**a’**), top right), lymphocytes ((**b’**), right; (**d’**)), and stromal cells (**a’**,**c’**). Bars: 100 µm.

**Figure 5:**
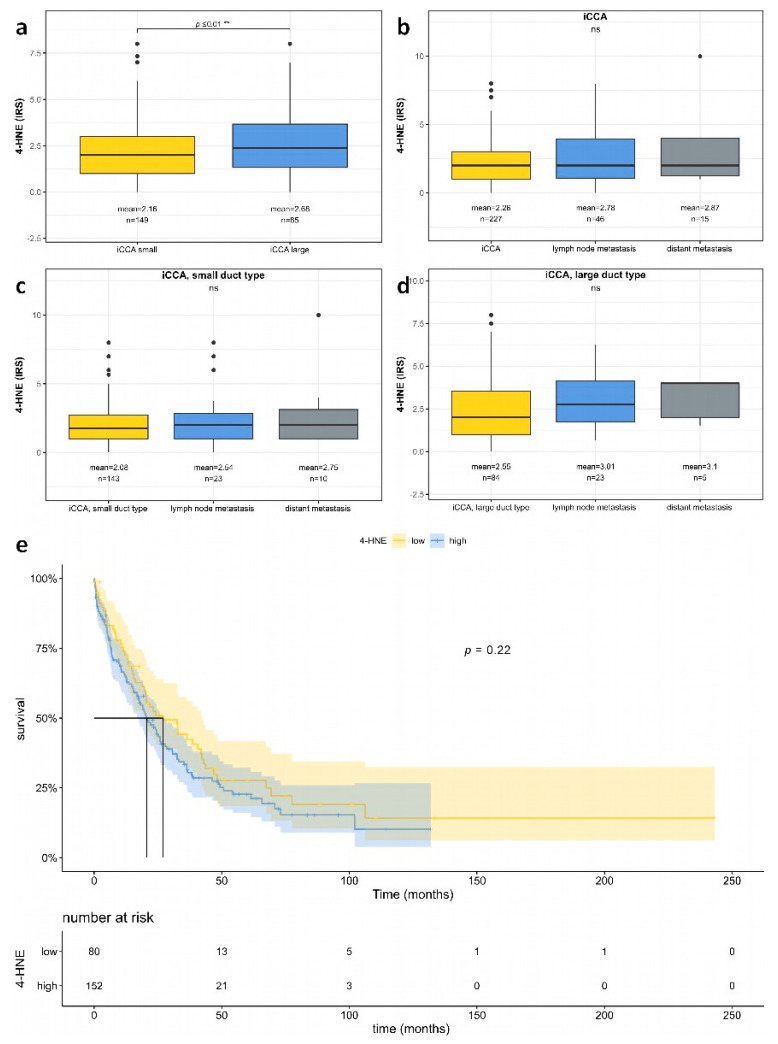
Semiquantitative analysis of 4-HNE in intrahepatic cholangiocarcinoma (iCCA) as analyzed by immunohistochemistry. (**a**) Boxplot analysis demonstrating significantly increased 4-HNE levels in large duct iCCA. (**b**–**d**) No significant differences in 4-HNE levels between primary iCCA and lymph node and distant metastases ((**b**): iCCA, all types; c: small duct iCCA; (**d**): large duct iCCA). (**e**) Kaplan–Meier plot shows no significant differences between iCCA with high and low 4-HNE expression. If a *p* value is less than 0.01, it is flagged with 2 stars (**). No significance is labeled as “ns”.

The strong correlation of 4-HNE levels, as tested by immunohistochemistry, with survival in HCC patients prompted the investigation of whether this relationship is a fundamental characteristic of primary liver carcinomas or a specific feature limited to HCC. To address this, we conducted a study on 294 iCCA cases from 236 patients within a clinically established cohort [29], examining their 4-HNE expression (Figure 4). No significant association was found between 4-HNE levels and the overall survival of iCCA patients (Figure 5e). Subgroup analysis did not reveal any significant difference in survival between small and large duct iCCA, although the large duct iCCA showed slightly higher 4-HNE amounts than the small duct iCCA (Figure 5a; for further details, see Supplementary Figure S1).

## 4. Discussion

The present study aimed to investigate the prognostic role of 4-HNE in liver cancer within a large cohort of 797 primary liver cancers, including 561 HCC and 236 iCCA. Our findings revealed distinct associations and prognostic implications of 4-HNE expression in HCC, while no significant correlations were found in iCCA.

We observed a significant decrease in 4-HNE levels during hepatocarcinogenesis, from non-neoplastic liver tissue to primary HCC, relapse tumors, as well as lymph node and distant metastases. This indicates the potential role of 4-HNE in HCC progression and metastasis. In this line, our survival analysis demonstrated that patients with low 4-HNE levels had significantly worse overall survival compared to those with high intratumoral 4-HNE adducts, highlighting the prognostic significance of 4-HNE in HCC. Further exploration of prognostic factors in HCC revealed intriguing correlations with 4-HNE levels. HCC with low intratumoral 4-HNE levels exhibited characteristics associated with aggressive tumor behavior, such as increased vascular invasion and poor tumor differentiation. However, these correlations may also represent dependent variables. To investigate this issue, i.e., to determine whether 4-HNE expression negatively affects patient outcomes independent of other prognostic factors, we performed a multivariate regression analysis. This analysis confirmed the independent prognostic significance of 4-HNE in HCC, paralleling established factors such as vascular invasion [30], BCLC stage [31], and serum alpha-fetoprotein (AFP) levels [32]. These data support the hypothesis that reduced lipid peroxidation, indicated by reduced 4-HNE levels, is associated with increased aggressiveness in HCC. In addition, a trend towards increased apoptosis as tested by caspase 3 was observed in HCC with higher 4-HNE levels, which reached no significance. Moreover, lower amounts of 4-HNE were detected in macrotrabecular-massive HCC, which constitutes an HCC subtype with known unfavorable outcomes [28], associated with cellular proliferation, activation of anti-apoptotic proteins, and an angiogenesis activation-related carcinogenesis pathway. These findings are noteworthy, as they may guide the development of distinct therapy regimes beneficial to patients suffering from macrotrabecular-massive HCC [33].

4-HNE originates from mitochondria through the process of lipid peroxidation, forming adducts with proteins, DNA, and lipids. Due to the crucial role of ROS, manipulating its mitochondrial generation has been proposed as a potential target for cancer prevention and therapy [34]. For example, doxorubicin, one of the most effective anticancer drugs, acts via ROS [35]. Other therapeutic targets under discussion include 4-HNE and the antioxidant MitoQ, which interferes with the regulation of ROS production and has implications for liver fibrosis development [36]. Elevated 4-HNE levels interact with mitochondrial proteins, contributing to the mutagenic and carcinogenic effects of lipid peroxidation [34]. Consequently, a promising therapeutic strategy involves targeting 4-HNE through the activation of the ALDH2 enzyme using Alda-1, which enhances its activity [37]. ALDH2 is known for its role in ethanol metabolism but also plays a key role in oxidizing lipid peroxidation products, like 4-HNE [38]. Inhibition of ALDH2 is a well-known pharmacological intervention as a treatment for alcohol abuse [39]. ALDH2 is activated by Alda-1, resulting in enhanced catalytic activity, and protects from nucleophilic attacks of reactive aldehydes, such as 4-HNE [40]. Hence, increased detoxification of 4-HNE by Alda-1 offers significant potential for altering ROS-mediated cellular damage and is thus a potential therapeutic agent to prevent ROS-associated diseases. Preliminary studies have shown promising results utilizing Alda-1 to augment 4-HNE detoxification in Alzheimer’s disease progression. The study demonstrated that treatment with Alda-1 effectively prevented the impairment of angiogenesis in cultured endothelial cells induced by amyloid β peptide, which is known to induce oxidative stress [41]. However, intriguingly, our study revealed that despite its association with cellular and DNA damage, lower levels of 4-HNE were paradoxically linked to a subgroup of HCC patients with a worse prognosis, raising questions about the underlying mechanisms driving this observation.

Furthermore, we observed a lower frequency of liver cirrhosis and, when present, a lower Child–Pugh score in the HCC group with lower intratumoral 4-HNE expression. It is important to note that our patient cohort consisted of resected HCC with available histology, as per treatment guidelines [42]. As a result, advanced HCC in palliative settings and patients with worse liver function were excluded from the study, which could potentially influence the generalizability of our findings.

Oxidative stress induced by ROS is a major mechanism leading to nonalcoholic fatty liver disease (NAFLD) [43]. Additionally, ROS also plays a pivotal role in alcoholic-induced steatosis (alcoholic fatty liver disease; AFLD) through impaired hepatic lipid metabolism [44]. However, the adaptive mechanisms involved in carcinogenesis and dedifferentiation also include oxidative stress mitigation, such as the metabolism of ROS and 4-HNE [45]. This detoxification occurs through reactions into less reactive products with glutathione, reduction to 1,4-dihydroxy-2-nonene, and oxidation to 4-hydroxy-2-nonenoic acid [34]. These mechanisms may explain the reduced levels of 4-HNE in patients with a worse outcome. Additionally, some tumors use ROS scavenging techniques to keep ROS levels low, enter a quiescent state, and thus resist systemic therapy more effectively [46,47]. Furthermore, cancer stem cells can produce vast amounts of glutathione to enhance the metabolism of ROS, reducing the levels of 4-HNE and improving their survivability against radio- and chemotherapy [45,48,49]. Sorafenib, one of the therapeutic options for advanced HCC, generates ROS, exacerbating oxidative stress within cancer cells. In sorafenib-resistant cells, increased UBQLN1 expression leads to the degradation of PGC1β through a ubiquitination-independent mechanism, resulting in reduced mitochondrial biogenesis and decreased ROS production [50]. These findings suggest that low 4-HNE levels characterize a specific subgroup of HCC with enhanced 4-HNE metabolism, a byproduct of systemic therapy, potentially leading to increased detoxification of lipid peroxidation intermediates. HCC with low levels of 4-HNE may, therefore, represent a subgroup of HCC that may be specifically resistant to treatment with sorafenib. Further studies are needed to corroborate this assumption.

Our study reveals an association between decreased levels of 4-HNE and unfavorable survival outcomes in HCC patients, which is in accordance with previous studies conducted on various types of cancer. For instance, in prostate cancer, decreased levels of 4-HNE have been linked to disease progression [51]. Similarly, decreased 4-HNE levels have been associated with breast cancer [52], gastric cancer [53], urothelial carcinoma [54], ovarian cancer [55], or with a worse prognosis, e.g., in lung squamous cell carcinoma [56]. Reduced levels of 4-HNE may therefore have implications for cancer development and progression. The decrease in intratumoral 4-HNE as tested by immunohistochemistry is paralleled by increased tumor growth and aggressiveness; however, further investigation is warranted to unravel the precise mechanisms through which 4-HNE influences tumor behavior and to explore its therapeutic implications for improving patient outcomes.

In a parallel study of mouse HCC induced by the potent hepatocarcinogen diethylnitrosamine, 4-HNE localized to frequent eosinophilic globules, which may correspond to so-called pale bodies/hyalin-like globules [57] in dysplastic foci, nodules, and small HCC, which were frequently found in mice but only to a minor percentage in human hepatocarcinogenesis. Yet, this finding may partially explain the higher levels of 4-HNE present in specific subtypes and less advanced HCC. The significance of pale bodies is currently unknown. In aged hepatocytes in zone 3, lipofuscin, a lipid degradation product, may be partially stained with antibodies against 4-HNE; however, we did not observe a significant correlation between 4-HNE staining and the age of the respective patient. 

In contrast to HCC, in iCCA, our analysis did not reveal any significant association between 4-HNE expression levels and overall survival. While large duct iCCA exhibited slightly higher 4-HNE expression compared to small duct iCCA, this difference was not statistically significant. The association between 4-HNE and patient survival observed in HCC may be specific to HCC rather than a universal characteristic of primary liver carcinomas.

## 5. Conclusions

In this study, we investigated 4-hydroxy-nonenal (4-HNE) in a large, clinically well-characterized patient cohort with primary liver carcinomas, including 561 hepatocellular carcinomas (HCC) and 236 intrahepatic cholangiocarcinomas (iCCA), using immunohistochemistry. 4-HNE is a key molecule in the complex molecular processes of cellular oxidative stress regulation and may act as a signal transducer, inducing inflammation. Both HCC and iCCA demonstrated differential 4-HNE expression in different tumor subtypes. We found a significant adverse association between low 4-HNE levels and overall survival in HCC but not in iCCA patients. This suggests that reduced lipid peroxidation in HCC may contribute to increased invasiveness and dedifferentiation or indicate the potential for tumor evasion of therapeutic approaches. Interestingly, we also observed that low levels of 4-HNE expression were associated with a higher number of vascular invasions, lower tumor differentiation, and a macrotrabecular-massive HCC subtype. These findings underscore the importance of elucidating the role of 4-HNE in HCC and investigating 4-HNE modulating agents as therapeutic agents, emphasizing the need for further comprehensive and mechanistic studies in this area.

## Figures and Tables

**Table 1:** Clinicopathologic characteristics of the HCC cohort and analyses of prognostic factors.

Characteristic		Low 4-HNE	High 4-HNE	*p*-Value *
		*n* = 424 (79%)	*n* = 112 (21%)	
Age at diagnosis [years] (range)		65.3 (57.8, 71.8)	60.4 (52.6, 67.0)	**<0.001**
Tumor max. diameter [mm] (range)		44 (26, 90)	32 (22, 50)	**<0.001**
Gender	male	334 (79%)	88 (79%)	0.9
	female	90 (21%)	24 (21%)	
Alcohol abuse	absent	305 (72%)	72 (64%)	0.12
	present	119 (28%)	40 (36%)	
HCV	absent	343 (81%)	86 (77%)	0.3
	present	81 (19%)	26 (23%)	
HBV	absent	346 (82%)	90 (80%)	0.8
	present	78 (18%)	22 (20%)	
NASH	absent	386 (91%)	108 (96%)	0.059
	present	38 (9.0%)	4 (3.6%)	
Hemochromatosis	absent	403 (95%)	108 (96%)	0.5
	present	21 (5.0%)	4 (3.6%)	
Cirrhosis	absent	170 (40%)	24 (21%)	**<0.001**
	present	254 (60%)	88 (79%)	
Child–Pugh score	A	160 (58%)	35 (38%)	**0.001**
	B	97 (35%)	41 (45%)	
	C	19 (6.9%)	15 (16%)	
BCLC Stage	A	72 (17%)	26 (23%)	**<0.001**
	B	278 (66%)	65 (58%)	
	C	55 (13%)	6 (5.4%)	
	D	19 (4.5%)	15 (13%)	
Grading	G1	74 (19%)	25 (26%)	0.2
	G2	214 (55%)	53 (55%)	
	G3	98 (25%)	18 (19%)	
VETC	negative	344 (81%)	97 (87%)	0.2
	positive	80 (19%)	15 (13%)	
Vascular invasion	absent	254 (60%)	91 (81%)	**<0.001**
	present	170 (40%)	21 (18%)	
Multifocality	absent	239 (65%)	53 (58%)	0.3
	present	131 (35%)	38 (42%)	
Macrotrabecular subtype	absent	379 (91%)	108 (98%)	**0.009**
	present	39 (9.3%)	2 (1.8%)	
GS overexpression	absent	340 (80%)	84 (76%)	0.4
	present	84 (20%)	26 (24%)	

* Wilcoxon rank sum test; Pearson’s Chi-squared test; Fisher’s exact test. NASH: nonalcoholic steatohepatitis; VETC: vessels encapsulating tumor clusters; GS: glutamine synthetase. The bold font indicates values that are statistically significant (*p* < 0.05).

## Data Availability

The datasets generated during and/or analyzed during the current study are available from the corresponding author upon reasonable request.

## Supplementary Materials

The following supporting information can be downloaded at: https://www.mdpi.com/article/10.3390/biomedicines11092471/s1. Figure S1: Box plot analysis of 4-HNE IRS and clinicopathological features in intrahepatic cholangiocarcinoma (iCCA).
