# Supplementary material for: Reduced Lipid Peroxidation Predicts Unfavorable Prognosis in Hepatocellular Carcinoma, but Not Intrahepatic Cholangiocarcinoma
Source: Biomedicines. 2023 Sep 6;11(9):2471. doi: 10.3390/biomedicines11092471 (PMC10525544; doi:10.3390/biomedicines11092471)
Supplement: Supplementary file 1 [file biomedicines-11-02471-s001.zip › biomedicines-2556889-supplementary.pdf]

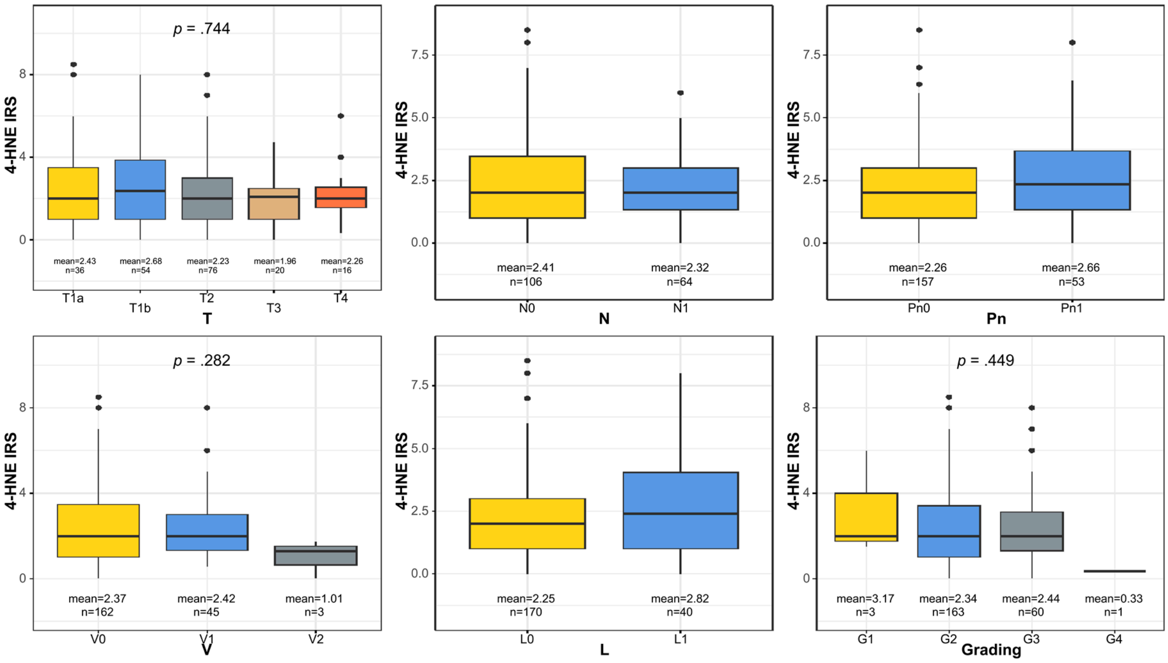

Figure S1: Box plot analysis of 4-HNE IRS and clinicopathological features in intrahepatic cholangiocarcinoma (iCCA).
